# Staging of Neurofibrillary Pathology in Alzheimer's Disease: A Study of the BrainNet Europe Consortium

**DOI:** 10.1111/j.1750-3639.2008.00147.x

**Published:** 2008-10

**Authors:** Irina Alafuzoff, Thomas Arzberger, Safa Al-Sarraj, Istvan Bodi, Nenad Bogdanovic, Heiko Braak, Orso Bugiani, Kelly Del-Tredici, Isidro Ferrer, Ellen Gelpi, Giorgio Giaccone, Manuel B Graeber, Paul Ince, Wouter Kamphorst, Andrew King, Penelope Korkolopoulou, Gábor G Kovács, Sergey Larionov, David Meyronet, Camelia Monoranu, Piero Parchi, Efstratios Patsouris, Wolfgang Roggendorf, Danielle Seilhean, Fabrizio Tagliavini, Christine Stadelmann, Nathalie Streichenberger, Dietmar R Thal, Stephen B Wharton, Hans Kretzschmar

**Affiliations:** 1 Department of Neuroscience and Neurology, Kuopio UniversityKuopio, Finland; 2 Center for Neuropathology and Prion Research, München Ludwig-Maximilians-University, MunichGermany; 3 Department of Clinical Neuropathology, Kings College Hospital and the Institute of PsychiatryKings College London, UK; 4 Department of Geriatrics, Karolinska InstitutetHuddinge, Sweden; 5 Institute fur Neuropathologie, Institute fur Clinical NeuroanatomyFrankfurt/Main, Germany; 6 Fondazione IRCCS Istituto Neurologico Carlo BestaMilano, Italy; 7 Institut de Neuropatologia, Universitat de BarcelonaBarcelona, Spain; 8 Institute of Neurology, Medical University of ViennaVienna, Austria; 9 MBG University Department of NeuropathologyHammersmith Hospitals Trust and Imperial College London, UK; 10 Academic Nit of Pathology Sheffield University Medical SchoolSheffield, UK; 11 Netherlands Brain BankAmsterdam, the Netherlands; 12 Department of Pathology, National and Capodistrian University of AthensAthens, Greece; 13 OPNI, National Institute of Psychiatry and NeurologyBudapest, Hungary; 14 Institute for Neuropathology, University of BonnBonn, Germany; 15 Université Lyon 1, Faculté de Médecine Laennec, Hospices Civils de Lyon, Centre de Pathologie et de Neuropathologie EstLyon, France; 16 Pathologisches Institut, Abteilung Neuropathologie der Universität WürzburgWürzburg, Germany; 17 Dipartimento di Scienze Neurologiche, Università di BolognaBologna, Italy; 18 Laboratoire de Neuropathologie Raymond Escourolle, Université Pierre et Marie Curie and INSERMParis, France; 19 UniversitätsklinikumGöttingen, Germany; 20 Institute of Pathology – Laboratory of Neuropathology, University of UlmUlm, Germany

**Keywords:** Alzheimer's disease, immunohistochemistry, neurofibrillary pathology, neuropathological diagnosis, BrainNet Europe consortium

## Abstract

It has been recognized that molecular classifications will form the basis for neuropathological diagnostic work in the future. Consequently, in order to reach a diagnosis of Alzheimer's disease (AD), the presence of hyperphosphorylated tau (HP-tau) and β-amyloid protein in brain tissue must be unequivocal. In addition, the stepwise progression of pathology needs to be assessed. This paper deals exclusively with the regional assessment of AD-related HP-tau pathology. The objective was to provide straightforward instructions to aid in the assessment of AD-related immunohistochemically (IHC) detected HP-tau pathology and to test the concordance of assessments made by 25 independent evaluators. The assessment of progression in 7-µm-thick sections was based on assessment of IHC labeled HP-tau immunoreactive neuropil threads (NTs). Our results indicate that good agreement can be reached when the lesions are substantial, i.e., the lesions have reached isocortical structures (stage V–VI absolute agreement 91%), whereas when only mild subtle lesions were present the agreement was poorer (I–II absolute agreement 50%). Thus, in a research setting when the extent of lesions is mild, it is strongly recommended that the assessment of lesions should be carried out by at least two independent observers.

## INTRODUCTION

Alzheimer's disease (AD)-related pathology, both neuritic plaques (NP) and neurofibrillary tangles (NFT) is commonly seen in aged subjects, both in neurologically unimpaired as well as in the demented, as was already reported by Tomlinson and colleagues in 1968 and 1970 (20,21). Consequently, before one can make a definite diagnosis of AD, certain clinical symptoms as well as disease-specific pathology should be present. The common view is that if one wishes to obtain an exact and reproducible diagnosis in an aged subject, this requires a detailed assessment of both clinical and neuropathological phenotype. This paper deals solely with the latter topic, that is, the neuropathological assessment of AD-related neurofibrillary lesions seen in aged and demented individuals.

There are many prerequisites before one can issue consensus recommendations for neuropathological diagnostic criteria. They have to be applicable for routine working conditions and also to be economical. They have to be widely acceptable, as well as reproducible and reliable for use, and moreover, they have to be comprehensible and simple. Furthermore, using current recommendations, rather than relatively nonspecific chemical stains, these kinds of criteria should have a molecular basis (1).

**Figure 1:**
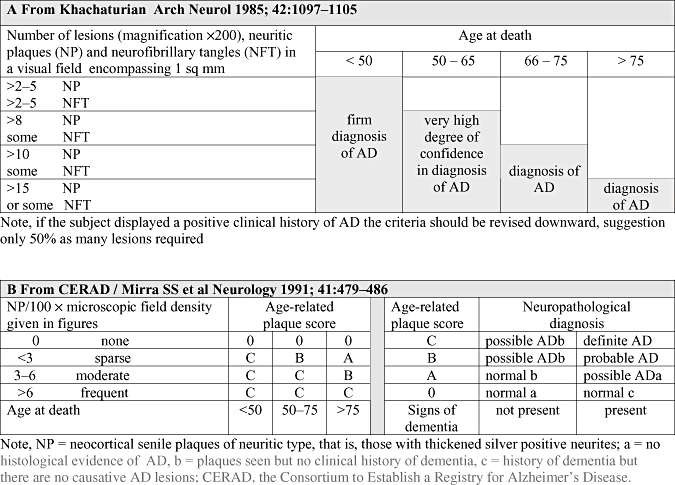
In both (A) and (B) the assessment of Alzheimer's disease (AD)-related pathology is based primarily on the counts of senile/neuritic plaques visualized by silver stains. As the formation of pathology spans decades and is also seen in the brains of unimpaired subjects to obtain a definite diagnosis of AD, both the age of the subject and the clinical symptoms are of significance.

The first published consensus guidelines regarding assessment of AD-related hallmark lesions, NP and NFT, were discussed in a workshop jointly sponsored by the National Institute of Aging, the American Association of Retired Persons, the National Institute of Neurological and Communicative Disorders and Stroke and the National Institute of Mental Health. As a result, Khatchaturian (9) published a report in 1985 where recommendations were issued regarding sampling of tissue, methods to be used, and the assessment of lesions (Figure 1A). The emphasis was placed on the quantitative assessment of both NP and NFT in relation to the age of the patient together with the clinical history that influenced the assessment outcome or diagnosis (Figure 1A). The quantification of the hallmark lesions, however, proved to be more complicated than expected. In 1990, a European multicentre study under the auspices of the European Community's Concerted Action Programme on Ageing and Diseases (EURAGE) reported that when assessing the lesions typical of AD numerous staining techniques were used by the 11 participating laboratories, and thus, it was not surprising that the concordance between 11 investigators assessing NP and NFT was poor (7). In the following year, the Consortium to Establish a Registry for Alzheimer's disease known by the acronym the CERAD, launched revised instructions (Figure 1B) (12). CERAD defined in more detail one of the hallmark lesions, that is, NP and narrowed down the section thickness. They also provided instructions regarding the staining to be used and a schematic presentation of the scoring of NP to facilitate reproducibility between different centers. The simplicity and clarity of these instructions meant that they soon acquired a wide number of users (12).

The main problem concerning the CERAD strategy is related to the methodologies being used. The recommended fluorescent thioflavine S preparation is viewed under ultraviolet light and thus requires special equipment, not always available in all laboratories. The recommended silver technique such as the modified Bielschowsky method is somewhat laborious and capricious, requires a well-trained technician and is influenced by ambient temperature (12). In view of this, our BrainNet Europe interlaboratory study confirmed that indeed the quality of produced silver stained sections varied significantly from center to center (2). Furthermore, to our surprise, not only the quality of stained sections varied, but also the quantitative results were not in full agreement when assessing the hallmark lesions of AD. The concordance of assessment of AD-related pathology following the instructions of the CERAD classification was also reported to be less than optimal (2, 13).

The study described above dealt with the assessment of hallmark lesions in the neocortex without paying any significant attention to their regional distribution. Already in 1977, Brun and colleagues reported that in AD, the degeneration of the brain seemed to display primarily a temporo-parietal pattern of distribution (8). This distinctive distribution of AD-related pathology was later confirmed and described in detail by Heiko and Eva Braak, this being currently referred to as Braak staging of AD-related NFT/neuropil thread (NT) pathology, an assessment strategy that also has been broadly accepted (3).

One of the problems with the Braak staging was that it was based on the use of 100-µm-thick sections, which are not practical for use in routine diagnostics, and thus, numerous modifications introduced by various centers have been published (6). Furthermore, the silver stains used to visualize NFT and NTs were recently shown by our BrainNet Europe interlaboratory study to yield poor reproducibility (2).

**Figure 2:**
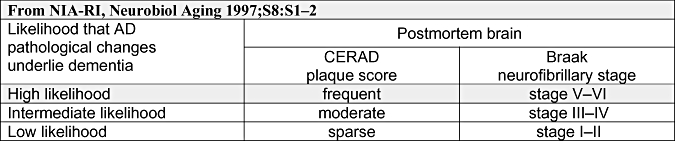
The diagnosis of Alzheimer's disease (AD) is given as a likelihood that both of the AD-related neuropathological lesions, neurofibrillary tangles and neuritic plaques, are causative regarding clinical symptoms. NIA-RI = the National Institute on Aging and Reagan Institute; CERAD = the Consortium to Establish a Registry for Alzheimer's disease.

Even though the methods were known to be both laborious and capricious, the currently commonly used consensus recommendations for post-mortem diagnosis of AD, launched by the National Institute on Aging and Reagan Institute (NIA-RI) working group in 1997, combined the two recommendations from 1991, CERAD classification and Braak staging (Figure 2) (19). In these NIA-RI recommendations, Braak stages (transentorhinal, limbic and isocortical) are combined with the NP scores according to CERAD (infrequent, moderate and frequent) resulting in a statement of likelihood (low, intermediate, high) that dementia is due to AD-related lesions. Thus, in NIA-RI recommendations, both the extent and distribution of the dual pathology are important, that is, there has to be both NFT and NP pathology. Additionally, it was stated that in AD research centers, specific immunohistochemical (IHC) stains should be used with the intention of correlating IHC stained AD-related lesions with the conventional stains that demonstrate these lesions.

Recently, it has been recognized that molecular classifications represent the future for neuropathological diagnostic work (1). Rather than using nonspecific chemical stains, IHC methods that are based on precise molecular components are clearly preferable when available.

Thus for the diagnosis of AD, one should assess the brains regarding the occurrence of both hyperphosphorylated tau (HP-tau) a protein which is found both in its soluble (pre-tangles and NTs) and insoluble (NFT and NTs) forms and also β-amyloid (Aβ) protein which is found as variform plaques in parenchyma and is present in cerebral amyloid angiopathy (CAA) in vessel walls.

Braak and colleagues reported in 2006 that Braak staging of AD-related neurofibrillary lesions can also be carried out on IHC stained conventional 5–7-µm-thick sections (6). The immunolabeled AD-related neurofibrillary lesions vary, however, from those seen when using the silver method, that is, Gallyas. The silver method stains insoluble fibrillary NFT whereas the IHC method labels the antigen, that is, HP-tau with both the soluble and insoluble forms of the protein. Thus, when one compares the two stainings, the extent of pathology is more pronounced when using IHC methods and furthermore the predominant lesion in IHC stained sections are NTs rather than NFT. Thus, the proposed staging of AD-related neuronal pathology using IHC methodology is primarily based on the regional distribution of stained NTs. The benefits of IHC methods in the assessment of AD-related NT pathology were also highlighted in the BrainNet Europe interlaboratory study which revealed that both the quality of staining as well as assessment of IHC stained sections yielded higher reproducibility when compared with silver stained sections (2).

In line with the above, the assessment of plaques applying IHC methodology has been recommended and moreover, instead of counting or assessing the Aβ burden it has been proposed that the presumed stepwise regional deposition of the Aβ, that is, aggregation phases 1–5, should be assessed (17).

As far as we are aware, there has never been a thorough assessment of the reproducibility of either neuronal or plaque-related IHC labeled pathology in AD. The objective of this study was to devise straightforward instructions for the assessment of IHC labeled AD-related NT pathology that could be followed by any neuropathologist, irrespective of his/hers familiarity with AD cases. Another objective was to test the feasibility of using the guidelines by conducting an interlaboratory experiment including 25 independent evaluators routinely handling brain samples obtained from subjects with neurodegenerative diseases.

## MATERIAL AND METHODS

**Figure 3:**
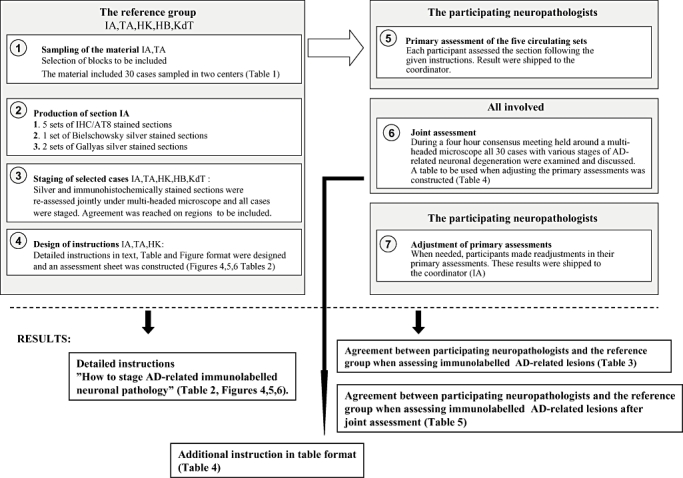
Flowchart delineating the structure of the study. IHC = immunohistochemistry; AD = Alzheimer's disease.

The general working order is summarized in Figure 3.

### Sampling of material

Thirty cases were included that were collected in two centers and the sampling of the blocks was carried out by two experienced neuropathologists (IA, TA). The cases were selected based on the Braak stage to which they were assigned by IA and TA after application of silver stain. The goal was to include all severities of the disease, that is, all the various stages of AD-related neurofibrillary pathology. The samples were taken for the routine diagnostics and obtained within a 10-year time span. The demographics of the subjects are given in Table 1. The selection of anatomical regions to be sampled was based on the requirements listed in current consensus criteria (NIA-RI), and was also influenced by known general practice among neuropathologists. The specimens included were the samples from middle frontal gyrus, inferior parietal lobule, superior and middle temporal gyrus, occipital cortex including calcarine fissure, posterior hippocampus at the level of lateral geniculate nucleus and anterior hippocampus at the level of uncus. A total of eight sets of 7-µm thick sections were produced from all six brain areas of the 30 cases.

**Table 1:** Demographics of the included cases. Abbreviations: CERAD = The Consortium to Establish a Registry for Alzheimer's disease; NP = neuritic plaques; NFT = neurofibrillary tangles; NIA-RI = The national institute on Aging and Reagan Institute working group; NT = neuropil threads; IHC = immunohistochemistry; HP-tau = hyperphosphorylated tau.

Case	Gender	Age at death	Brain weight	Clinical symptoms of dementia (yes/no)	CERAD NP counts in Bielschowsky	Braak stage based on NFTs in Gallyas	NIA-RI likelihood when applicable	Braak stage based on NTs in IHC/HP-tau
1	Male	46	1230	No	None	0	No	0
2	Female	39	1175	No	None	0	No	+
3	Male	71	1400	No	None	1	Not applicable	1
4	Male	77	1410	No	None	1	Not applicable	1
5	Female	89	1220	No	None	1	Not applicable	1
6	Female	81	1110	No	None	1	Not applicable	1
7	Female	85	1245	No	None	1	Not applicable	1
8	Female	88	1100	No	Moderate	2	Not applicable	2
9	Male	80	1465	No	None	2	Not applicable	2
10	Female	89	1170	Yes	Sparse	2	Low	2
11	Female	82	1290	No	None	2	Not applicable	2
12	Female	86	1045	No	Sparse	2	Low	2
13	Female	78	1330	Yes	None	2	Not applicable	2
14	Female	78	1080	No	Sparse	2	Low	2
15	Male	90	1400	Yes	Moderate	3	Intermediate	3
16	Male	79	1480	Yes	Moderate	3	Intermediate	3
17	Female	85	1250	No	Moderate	3	Intermediate	3
18	Male	76	1555	Yes	Sparse	3	Not applicable	3
19	Female	87	1030	No	None	3	Not applicable	3
20	Female	80	1180	No	None	3	Not applicable	3
21	Female	83	1200	Yes	Moderate	3	Intermediate	3
22	Male	83	1180	Yes	Moderate	4	Intermediate	4
23	Female	86	1095	Yes	Sparse	4	Not applicable	4
24	Female	85	1250	Yes	Frequent	4	Not applicable	4
25	Female	86	1200	Yes	Frequent	5	High	5
26	Female	81	1365	Yes	Frequent	5	High	5
27	Female	97	na	Yes	Moderate	5	Not applicable	5
28	Female	88	1180	Yes	Frequent	6	High	6
29	Male	76	1100	Yes	Frequent	6	High	6
30	Female	79	1190	Yes	Frequent	6	High	6

### Immunohistochemistry

Five sets of sections were manually stained applying IHC methodology. Shortly, after rehydration, the sections were incubated overnight at 4°C with a monoclonal primary antibody directed against HP-tau (Innogenetics Br-03, clone AT8, dilution 1:500) and the reaction product was visualized using the Zymed Lab-SA detection system (Zymed, San Fransisco, CA, USA) with the use of Biosource Romulin AEC as chromogen (Biocare medical, Walnut Creek, CA, USA).

### Conventional stainings

Two sets were stained applying Gallyas and one set was stained applying modified Bielschowsky silver impregnation technique.

### Reference assessment

**Figure 4:**
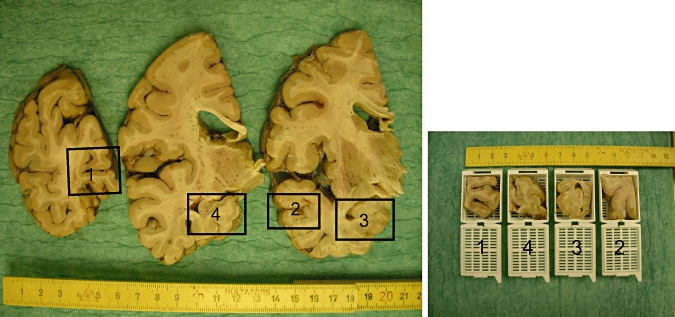
Gross view of the neuroanatomical regions included. 1-occipital cortex including calcarine fissure; 2-temporal cortex including middle temporal gyrus and at least a part of superior temporal gyrus, 3-anterior hippocampus at the level of uncus and 4-posterior hippocampus at the level of lateral geniculate nucleus.

The members of the reference group (IA, TA, HK, HB, KdT) jointly reassessed and staged all thirty cases around a multi-headed microscope (Table 1). The cases were first assessed in silver stains followed by a detailed analysis of the immunolabeled sections. Each case was given a Braak stage based on the assessment of Gallyas stained sections (3). The counts of NP were evaluated on Bielchowsky silver impregnated sections as described in the CERAD recommendations (12). When applicable, that is, Braak stage in agreement with CERAD stage, each case was given a NIA-RI likelihood statement (NIA-RI) (19). The typical features seen in IHC stained sections for each stage were agreed upon and these features were pinpointed in the final instructions. During the assessment of 30 cases, it was noted that the samples from middle frontal gyrus and inferior parietal lobe did not alter the staging, and thus, these samples were excluded. The samples included in this interlaboratory staging trial are superior and middle temporal gyrus, occipital cortex including calcarine fissure, posterior hippocampus at the level of lateral geniculate nucleus and anterior hippocampus at the level of uncus; they are shown in Figure 4.

### Instructions

**Figure 5:**
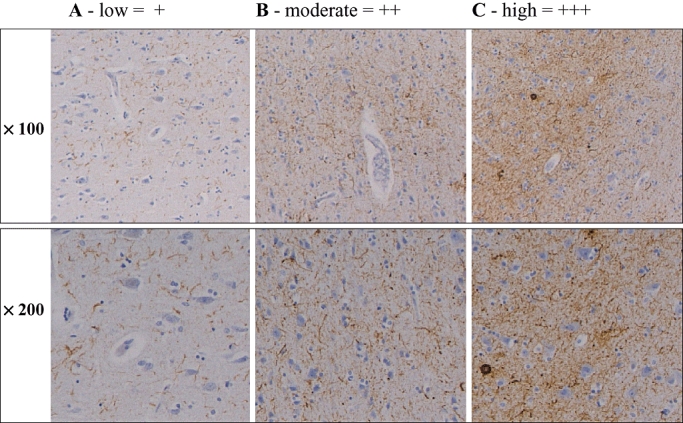
*Density of AT-8 immunopositive neuropil threads at magnifications × 100 and × 200*. A. Low (+), that is, immunoreactive (IR) structures are barely noted at low magnification. B. Moderate (++), that is, IR structures are easily seen at both magnifications. C. High (+++), that is, IR structures are seen even without the microscope. Sections are taken from occipital cortex.

**Figure 6:**
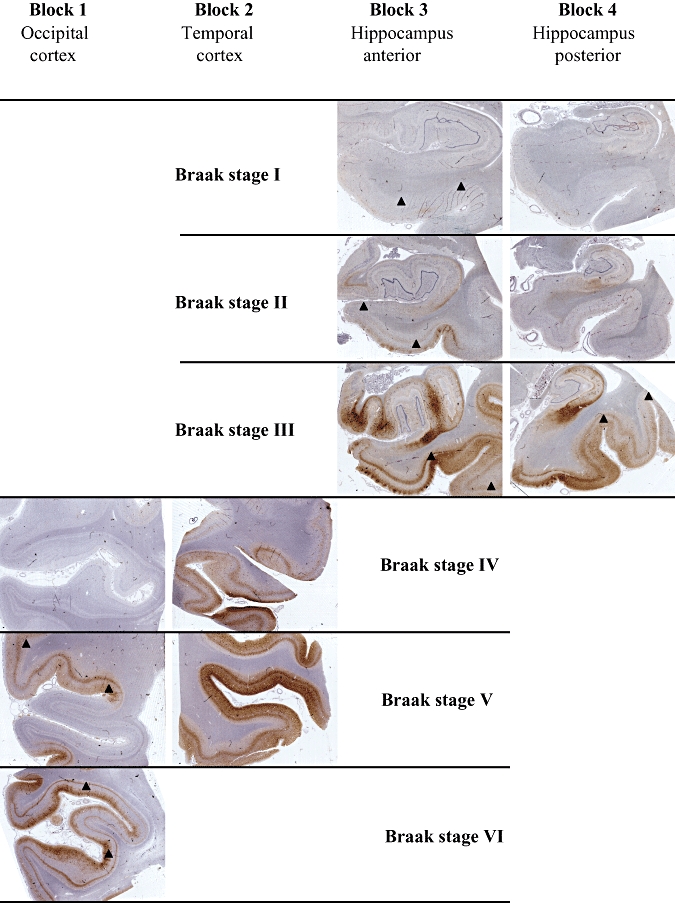
*Scanned immunohistochemically stained sections applying AT8 antibody*. Section from: Block 1 – occipital cortex including calcarine fissure; Block 2 – temporal cortex including middle temporal gyrus and at least a part of superior temporal gyrus; Block 3 – anterior hippocampus at the level of uncus; and Block 4 – posterior hippocampus at the level of lateral geniculate nucleus. The regions are given from left to right in the suggested order of assessment. The arrowheads indicate borders for the relevant neuroanatomical regions for each given stage: Braak I – transentorhinal region; Braak II – entorhinal region; Braak III – temporo-occipital gyrus; Braak IV – temporal cortex; Braak V – peristriatal cortex; and Braak VI striatal cortex.

Detailed assessment instructions were written by three members of the reference group (IA, TA, HK). The instructions included a detailed description of the samples (Table 2A, Figure 4), a description of the pathology to be assessed (Table 2B, Figure 5), and exact guidelines about the staging (Table 2C, Figure 6) which were based on the distribution and load of immunolabeling in various neuroanatomical regions. It should be stressed, as has been previously described (6) that the staging of the pathology was based on labeled NTs rather than NFT.

**Table 2:** Detailed instructions to be followed when assessing AD-related pathology (A) neuroanatomical regions (B) scoring of IHC labeling and (C) staging of AD-related pathology. Abbreviations: IHC = immunohistochemistry; AD = Alzheimer's disease; NT = neuropil threads.

(A) Harvested brain samples see also Figure 5			Main stage to be viewed
Section 1	The visual cortex including the calcarine fissure	The section contains the striatal area (Brodmann area 17 = the primary visual cortex with macroscopically identifiable band of Gennari) and para-/peristriate areas (Brodmann area 18/19 = a six layered cortex area which does not contain the band of Gennari).	AD stages VI and V
Section 2	The middle temporal gyrus	The section occasionally includes a portion of the superior temporal gyrus.	AD stage IV
Section 3	The anterior hippocampus and/or amygdala at the level of uncus	The section contains the parahippocampal gyrus with (trans-) entorhinal region and part of the occipito-temporal gyrus (=fusiform gyrus).	AD stages III, II and I
Section 4	The posterior hippocampus at the level of the lateral geniculate nucleus	The section includes posterior portions of the parahippocampal gyrus with varying remnants of the (trans-)entorhinal region or lingual gyrus. In most cases, the adjoining occipito-temporal gyrus (=fusiform gyrus) can also be seen.	AD stages II and III

Recommendation: Start viewing with the occipital section, followed by the temporal section, then move to the anterior section of the hippocampus, and conclude with the hippocampal section taken at the level of the lateral geniculate nucleus			

(B) Scoring of immunohistochemically labeled NTs, that is, density of IHC/AT8 positive NTs, see also Figure 6			

+	Barely present at all at ×100		
++	Easily noted at ×100		
+++	Can be visualized even without a microscope		

(C) Staging of AD-related neuronal pathology assessing IHC/AT8 stained NTs, see also Figure 6			

Stage VI	Severe involvement of occipital cortex	Immunopositive NTs of at least moderate density (++ or +++) in layer V of the striate area. This stage is so clearly visible that it may well be seen by the naked eye.	
Stage V		Immunopositive NTs of at least moderate density (++ or +++) in the superficial and deep layers of the peristriate (and often also parastriate) area.	
Stage IV	Severe involvement of the middle temporal gyrus	Immunopositive NTs of at least moderate density (++ or +++) in the superficial and/or deep layers of the middle temporal gyrus.	
Stage III	Involvement of posterior hippocampus at the level of the lateral geniculate nucleus	Immunopositive NTs of at least moderate density (++ or +++) in the outer and inner layers of remnants of the entorhinal region, continuing into the neocortex of the adjoining occipito-temporal gyrus.	
	Involvement of the anterior hippocampus at the level of uncus	Immunopositive NTs of at least moderate density (++ or +++) in the superficial and deep layers of the occipito-temporal gyrus. The immunopositivity in even a small region of the occipito-temporal gyrus adjoining the transentorhinal region means that the specimen should be diagnosed as stage III.	
Stage II	Involvement of posterior hippocampus at the level of the lateral geniculate nucleus	Immunopositive NTs of at least moderate density (++ or +++) in the outer layers of remnants of the entorhinal region and of at least low density (+ or ++ or +++) in the inner layers of remnants of the entorhinal region.	
	Involvement of the anterior hippocampus at the level of uncus	Immunopositive NTs of at least moderate density (++ or +++) in the outer layers of the entorhinal region and of at least low density (+ or ++ or +++) in the inner layers of the entorhinal region.	
Stage I	Involvement of the anterior hippocampus at the level of uncus	Immunopositive NTs of at least low density (+ or ++ or +++) are localized in the transentorhinal region.	
Stage +	Any section	a)Single or few immunopositive cell bodies (tangles or pre-tangles) in any or more than one region with distribution pattern that does not fit to one of the known tauopathies and/or	
		b)NTs, either scattered or of low density (+) in any or more than one region and their distribution pattern does not fit to one of the known tauopathies and	
		c)criteria for AD-related neuronal pathology stages 0, I to VI are not fulfilled.	
Stage 0	All sections	To award a diagnosis of stage, 0, all four sections must be IHC/AT8 negative.	

Note, immunoreactive cell bodies (tangles or pre-tangles) may be seen in various hippocampal regions or in the neocortex at all stages and even in stage +, but this should not influence the decision-making about the staging. The crucial feature is the presence or absence of IHC/AT8-labeled NTs.

### BNE participant efforts

Twenty-five participants assessed and staged each case as instructed. The results were recorded on the assessment sheets which were sent to the coordinating center. These assessment sheets included information on whether or not the participant had identified the required neuroanatomical regions, the actual assessment of labeled NTs and NFT and their designated staging of the case.

### Consensus meeting and joined assessment

A joint assessment of IHC labeled sections of cases ranging from Braak stage 0 to VI was carried out around a multi-headed microscope. The diagnostic features of each stage were discussed and possible pitfalls were sought. Issues such as whether or not all cases fulfilled staging requirements, that is, typical vs. atypical cases were debated. In typical cases, the topography of labeled NTs was in compliance with the Braak hierarchical sequence of involvement of brain regions whereas in atypical cases this was not seen. Furthermore, it was discussed whether there was a need for modification of the originally issued instructions.

### Revision of the original assessment

After the joint assessment, all participants were instructed to adjust, if necessary, their originally designated stages. The final stage was supposed to be given based on the results found in the assessment sheets and following in detail the provided instructions. To help the participants in this task, IHC stained sections of all cases were scanned and a file with pictures was shipped to all participants. In addition, each participant was asked to assess whether or not the case was typical or atypical, that is, did or did not the distribution pattern of IHC labeling seem to follow the predicted sequence described by Braak and Braak (3). These revised assessments were then collected and the results were filed for analysis.

### Photomicrograph

Digital images were taken using a Leica DM4000 B microscope equipped with a Leica DFC 320 digital camera.

## RESULTS

The brain samples in this study were manually harvested by two neuropathologists over quite an extensive time period, and thus the sampling of the material resembles a real life scenario. As expected, some variation in the levels of sectioning was seen. However, this variation was not considered by the reference group, to be of significance with respect to the staging of IHC/NT pathology.

The results from the first round, prior to the consensus meeting, obtained when 25 neuropathologists assessed and staged the IHC stained sections of the 30 test cases adhering to the instructions are listed in Table 3. The agreement with the reference assessment was highest in the most severely affected cases (Braak stage VI-96%) and lowest in the mildly affected cases (Braak stage I-33%), overall 59%. There was 81% agreement with respect to the reference assessment for the isocortical Braak stages (V–VI), 68% for the limbic stages (III–IV) and 43% with respect to the entorhinal stages (I–II). In three cases (cases 3–5), the majority of the assessors disagreed with the reference assessments. All these cases were in stage I according to the reference assessment.

**Table 3:** Stages of AD-related neurofibrillary pathology when 25 neuropathologists have assessed immunohistochemically stained sections. The stage assessed by the reference group is given in bold. Abbreviations: ABS% = absolute agreement in percentage, that is, number of evaluation staged equally by the reference group and the 25 participating assessors; AD = Alzheimer's disease.

Case	0	+	I	II	III	IV	V	VI	ABS%*	ABS%†	ABS%‡	ABS%§
1	**13**	11	1						52	**42**	**42**	
2	17	**8**							32			
3	3	14	**8**						32			
4	3	16	**5**	1					20			
5	2	16	**3**	3			1		12	**33**		
6		4	**13**	7		1			52			
7	2	6	**12**	4		1			48			
8		3	5	**10**	6	1			40		**43**	
9		2	6	**12**	4	1			48			
10		2	6	**15**	1	1			60	**51**		
11	2		3	**17**	2		1		68			
12			1	**12**	10	1	1		48			
13		4	1	**11**	6	2	1		44			
14			5	8	**10**	2			40			
15			2	5	**14**	3	1		56			**59**
16				4	**16**	4	1		64			
17					**19**	5	1		76	**62**		
18				6	**17**		2		68		**68**	
19				9	**14**	2			56			
20				9	**15**		1		60			
21				4	**19**	1	1		76			
22					5	**19**	1		76			
23						**24**	1		96	**83**		
24				1	1	**19**	4		76			
25							**18**	7	72			
26							**17**	8	68	**63**		
27							**12**	13	48		**81**	
28							3	**22**	88			
29								**25**	100	**96**		
30								**25**	100			

* Percentage for each individual case.

† Percentage for all cases within a Braak stage.

‡ Percentage for cases within transentorhinal, limbic and isocortical stages.

§ Percentage for all.

Most of the cortical regions (striatal, peristriatal and temporal cortices) had been recognized with the exception of striatal and peristriatal cortices in case 28. The greatest difficulties had been encountered in identifying the outer layer of the entorhinal region (cases 5, 10, 23, 25), the inner layer of the entorhinal region (case 30) and the temporo-occipital region (case 18).

The labeled neurons, pre-tangles and tangles were recognized by all assessors whereas some variability was noted with regard to the assessment of the extent of labeled NTs. Overall, out of the 30 cases, an absolute agreement regarding the labeling intensity of NTs was reached in 57% for striatal cortex, in 50% for peristriatal cortex and in 17% for temporal cortex. Even in the remaining regions, no full agreement was reached and the assessment of labeling of NTs most frequently varied from none to low or from moderate to high. All possible ratings were given in three cases when the specimens included striatal cortex (cases 26, 27, 29), in five cases of peristriatal cortex (cases 25, 26, 27, 29, 30) and in one case where there was temporal cortex (case 25). The tendency to rate differently the extent of NTs was quite high in the regions of anterior hippocampus. In 15 out of 30 cases, all possible ratings were given for pathology present in the inner layer of the entorhinal cortex. A similar trend was seen in the outer layer of the entorhinal cortex (14/30), as well as in occipito-temporal gyrus and transentorhinal cortex (11/30 in both).

Most of the assessors participated in the consensus meeting where there was a joint assessment of all cases. It became evident that most of the assessors had not evaluated the stained sections with the naked eye (as recommended in the original instructions), though that is clearly useful method when assessing density of labeled NTs. High to moderate labeling of NTs is visible with naked eye inspection. Furthermore, it was noted that some assessors had found it difficult to follow the instructions. Thus some modifications, that is, clarifications of the original instructions were needed, and consequently, a new table was devised (Table 4). All cases were scanned and a file with pictures of the four stained sections was created to support the adjustment of stages if needed.

**Table 4:** Staging of Alzheimer's disease-related neuronal pathology on the basis of density and regional distribution of IHC/AT8 immunopositive neuropil threads. The density is scored on a four step scale 0-none; +-some; ++-moderate; +++ severe (see also Figure 6). Abbreviations: IHC = immunohistochemistry.

Section number	Region		Stage I	Stage II	Stage III	Stage IV	Stage V	Stage VI
Occipital cortex	Area 17		0–+	0–+	0–+	0–+	0–+	**++–+++**
	Area (18)/19		0–+	0–+	0–+	0–+	**++–+++**	++–+++
Temporal cortex			0–+	0–+	0–+	**++–+++**	++–+++	++–+++
Anterior hippocampus	Occipito-temporal gyrus		0–+	0–+	**++–+++**	++–+++	++–+++	++–+++
	Entorhinal region	Outer layers	0–+	**++–+++**	++–+++	++–+++	++–+++	++–+++
		Inner layers	0–+	**+–+++**	++–+++	++–+++	++–+++	++–+++
	Transentorhinal region		**+–+++**	++–+++	++–+++	++–+++	++–+++	++–+++
Posterior hippocampus	Occipito-temporal gyrus		0–+	0–+	**++–+++**	++–+++	++–+++	++–+++
	Remnants of the entorhinal region	Outer layers	0–+	**++–+++**	++–+++	++–+++	++–+++	++–+++
		Inner layers	0–+	**+–+++**	++–+++	++–+++	++–+++	++–+++

In stages I to VI various numbers of IHC/AT8-labeled neurons are seen, but the presence of IHC/AT8-positive neurons is not used as a diagnostic criterion. The hallmark lesion for each Braak stage is given in bold.

The results obtained when 25 neuropathologists adjusted their staging based on previously given assessments (ie, assessment sheets) and with the help of the scanned pictures are found in Table 5. Only in three cases, all in stage I according to the reference group (cases 3, 4 and 5), did the assessors mostly disagree with the reference assessment, whereas in general, the agreement with the reference assessment was 65%. In particular, the over-assessments seen in the original staging that primarily was based on assessment of NFT rather than NTs had mostly disappeared. The best results were obtained with stages where cortical regions were affected (stage VI = 100%, stage V = 81% and stage IV = 84%). The poorest results (42%) were noted for stage I which does require good familiarity with some neuroanatomical regions such as the entorhinal and transentorhinal regions. The agreement with the reference assessment was 91% for the isocortical stages (V–VI), 72% for the limbic stages (III–IV) and 50% for the entorhinal stages (I–II).

**Table 5:** Stages of AD-related pathology when 25 neuropathologists adjusted their original assessment after consensus meeting. The stage assessed by the reference group is given in bold. Abbreviations: ABS% = absolute agreement in percentage, that is, number of evaluation staged equally by the reference group and the 23 participating assessors; AD = Alzheimer's disease.

Case	0	+	I	II	III	IV	V	VI	ABS%*	ABS%†	ABS%‡	ABS%§
RZ146	**13**	11	1						52	42	42	
RZ176	17	**8**							32			
RZ92	6	11	**8**						32			
RZ165	5	13	**7**						28			
01–104	2	15	**7**	1					28	42		
97–147		3	**15**	7					60			
02–277	2	6	**15**	2					60			
00–255			5	**14**	6				56		50	
01–74		1	3	**17**	4				68			
02–96		2	9	**13**	1				52	57		
02–149	3	1	3	**16**	2				64			
070–00B		2	1	**11**	10	1			44			
093–02B		1	3	**15**	4	2			60			
RZ57			4	6	**14**	1			56			
03–170			3	3	**14**	5			56			65%
RZ239				6	**17**	2			68			
97–284				1	**23**	1			92	67		
02–031				5	**18**	1	1		72		72	
01–178				8	**17**				68			
RZ147			1	9	**15**				60			
04–025			1	7	**16**	1			64			
RZ181					11	**14**			56			
02–020						**25**			100	84		
RZ163						**24**	1		96			
03–212							**23**	2	92			
RZ153							**22**	3	88	81		
RZ162							**16**	9	64		91	
02–310								**25**	100			
RZ66								**25**	100	100		
99–156								**25**	100			

* Percentage for each individual case.

† Percentage for all cases within a Braak stage.

‡ Percentage for cases within transentorhinal, limbic and isocortical stages.

§ Percentage for all.

The reference group considered 28 out of the 30 included cases as typical with respect to the distribution of NT pathology, that is, distribution of pathology as expected for an AD stage. One case (case 1) lacked any pathology and in one case (case 2) only scattered labeled NFT were seen. In agreement with this, most cases were assessed as being typical AD cases also by most of the assessors, but there were some exceptions. Cases 8, 9, 10, 13 and 14 were considered as atypical by 30% of the assessors. In one of these “atypical” cases, the stages assigned varied from + to stage IV. In addition, cases assessed by most assessors as “+” (cases 3, 4 and 5) were considered as atypical, that is, the lesions were not considered to display a typical distribution pattern described for AD.

## DISCUSSION

It has recently been recognized that molecular classifications must be used in neuropathological diagnostics and thus IHC methods that are based on precise molecular components are recommended when available (1). With respect to AD, the assessment of occurrence of HP-tau seen in pre-tangles, tangles, neurites and NP and the assessment of the occurrence of Aβ in the parenchyma as variform plaques or as CAA is the diagnostic mode of choice. To our knowledge, there has never been a thorough evaluation of the reproducibility of the assessment of IHC labeled HP-tau or Aβ pathology in subjects with AD. This report deals with the reproducibility of evaluation when 25 neuropathologists assessed the HP-tau-related pathology in 30 subjects with various degrees of severity of AD-related neurofibrillary pathology.

The most important and also the most difficult task when staging lesions that progress in a continuous rather than stepwise manner is to define the cut off points, that is, the most typical features of hallmark lesions for each proposed stage. If one wishes to avoid the situation where the assessor has the impression that a case displays features seen in adjoining stages, the assessment instructions have to be described succinctly but clearly, even if they are based on arbitrary decisions. The emphasis should be on the most notable feature for each stage, that is, there should be a clear separation on one hand of what is or can be seen and on the other hand what is requested to be seen in each separate stage. The latter specification “what is required to be seen” is the pathognomonic feature of a given stage.

Instructions for the assessment in this trial were designed with three major parameters in mind. First, the region of significance in each stage was defined, that is, stage I – transentorhinal region, stage II – entorhinal region, stage III – temporo-occipital cortex, stage IV – temporal cortex, stage V and VI – occipital cortex. Second the type of lesion to be assessed was defined, that is, the NT network, with the third parameter being the intensity of staining of NTs, that is, the labeling had to be intense, that is, notable with naked eye inspection. In summary, there are two minimal requirements for the assessment of AD-related IHC labeled NT pathology: (i) adequate sampling of tissue blocks; and (ii) equally important is high quality IHC staining.

Twenty-five neuropathologists assessed 30 cases following instructions devised for this trial and reached a general agreement of 59%, ranging from 42% to 96% for different stages of progression of NT pathology, with the highest agreement achieved in the most severely affected cases. This agreement was improved to 65%, ranging from 42% to 100%, after a consensus meeting including a joint assessment of cases and a revision of the instructions.

One of the major oversights noted during the first assessment was that the labeled NFT were considered to be of significance even though the evaluators had been instructed otherwise. It seemed that it was difficult for a neuropathologist to stage a case as being in stage II–III, if NFTs were observed in temporal or occipital cortices. Thus the concordance of the assessments improved, simply by making the appropriate revision, that is, when staging IHC labeled section, the emphasis should be on the labeled NTs rather than on NFT.

As predicted, when assessing the highest stages of AD-related NT pathology, the agreements were close to excellent. The excellent agreement regarding AD-related NT pathology in stages IV–VI following our instructions indicates that indeed AD-related IHC labeled NT pathology is comparable even when assessed by different neuropathologists. In other words, a subject evaluated as being in stage V in one center will receive the same stage assessment in another center. This information is of importance not only from a diagnostic point of view but also regarding research situations. The high concordance in assessments makes it possible to combine post mortem brain material obtained from various centers. This will make it possible to conduct large-scale biochemical and other studies including hundreds of well-characterized samples. Furthermore, extensive screening of possible risk factors including hundreds, if not thousands, of neuropathologically verified AD patients can be carried out.

More disturbingly in the mildly affected cases, the agreement was poorer. One explanation for the poor agreement in stages I–III might be the prerequisite of good familiarity with the neuroanatomy of the hippocampal region, and particularly, the anterior part of hippocampus.

The lack of familiarity with the neuroanatomy of anterior hippocampus is probably due to the fact that most diagnostic neuropathologists tend to routinely sample posterior hippocampus at the level of the geniculate body. Minor parts of anterior hippocampus might be found in the often routinely sampled section of basal forebrain that also includes the amygdaloid nucleus.

Recent reports, have, however, emphasized that the transentorhinal and entorhinal regions seen in the anterior parts of hippocampus are of importance when assessing aged subjects and patients with mild memory impairment and thus a section taken from anterior part of hippocampus is recommended to be sampled routinely (3, 4, 6). Furthermore, with respect to AD pathology, one has to be able to identify the transentorhinal region in order to assess stage I, the entorhinal region in order to assess stage II, and the temporo-occipital regions have to be identified if one wishes to assess stage III. It is noteworthy that in the posterior parts of hippocampus, sampled at the level of lateral geniculate body, only remnants of transentorhinal cortex might be seen making the assessment of stage I virtually impossible. Similarly, the outer layer of entorhinal cortex might well be lost, complicating the assessment of stage II. In general, areas such as transentorhinal cortex as well as entorhinal cortex with its upper and inner layers might easily be overlooked if the sections are not optimally harvested. It is noteworthy that reliable identification particularly of the transentorhinal region in the section of anterior hippocampus or identification of the remnants of transentorhinal region in the section of posterior hippocampus in routine 5–7-µm-thick sections might sometimes be difficult even for a trained neuroanatomist or neuropathologist.

Moreover, it should be noted that the identification of hippocampal regions might have been easier for the assessors if all of the cases had been harvested by the same neuropathologist and within a shorter time span (sampling more homogenous). Sampling of brain specimens is, however, manual work, and thus, a wide range of variation in sectioning levels, is generally seen.

In some of our cases when only mild pathology was seen, the evaluator encountered difficulties in clearly stating, that the case represented stage I. Thus, the assessor tended to label these cases as “+”, that is, mild immunoreactivity present but criteria for AD-related neuronal degeneration are not fulfilled. This was probably due to the fact that they had not reliably identified the transentorhinal region. These cases were reassessed by the reference group and it was noted that indeed the transentorhinal and/or remnants of transentorhinal region required to be identified in stage I, were not always easily and reliably identified in the 7-µm-thick IHC stained sections. When these cases that were assessed by the majority as being “+” cases were excluded (case 3, 4, 5) the overall agreement reached a level of 67%.

An interrater-intrarater study assessing staging of AD-related NFT pathology has already been carried out in 1997 (14). The agreement between the six examiners including Heiko Braak, was almost perfect, kappa statistic values reaching values above 0.9. It is noteworthy, that the study was carried out on 100-µm-thick silver stained section, a method not practical under routine working conditions. Furthermore, it is not clear whether there were differences in the assessment of low vs. high stages. Consequently, comparison of results from 1997 with the results obtained in our study carried out applying IHC and 7-µm-thick sections and including 25 observers is not possible.

The poor agreement found in our study regarding mild involvement of AD pathology and employing 7-µm-thick sections emphasizes how important it is that when carrying out assessment of normal aged patients with mild cognitive impairment, that the sampling of tissue for neuropathological assessment is standardized. This is important if one intends to identify the transentorhinal region in 5–7-µm-thick routine sections. Furthermore, in research settings, it would probably be advisable that rather than collecting assessments carried out by different assessors, a reassessment of cases should be carried out, preferably by two independent neuropathologists well-familiarized with the anatomical structure of the anterior part of the hippocampus. In addition, when assessing AD cases one should always conduct a parallel assessment of the concomitant Aβ pathology. Instead of merely confirming that Aβ aggregates are seen, a staging of Aβ aggregates into phases based on regional distribution should be carried out, as has been described by Thal and colleagues in 2002 (17). The evaluation of the regional distribution of both NTs and Aβ might significantly influence the final assessment results.

Recently, McKee and colleagues reported that the visual association cortex, Brodmann area 19 (parastriatal cortex), was affected in the preclinical stage of AD (11). The area they assessed was described to be located laterally from calcarine cortex, striatal cortex (area 17) and peristriatal cortex (area 18). They reported that they had examined 25 neurologically unimpaired subjects in Braak stages ranging from I to IV whereas the CERAD NP counts ranged from none to frequent. Interestingly, in 13 of these cases, IHC labeled NFT were seen in area 19 ranging from one NFT/20× field to >10NFT/field (×200 magnification), whereas only occasional NFT were seen in area 17/18. The results of McKee and colleagues challenge the general view described by Heiko and Eva Braak that AD lesions develop according to a recognized and predictable sequence. When assessing our 30 cases, many of them were in the transentorhinal and limbic stages, IHC labeled NFT were occasionally (10 out of 24 cases) seen in areas 17/18, in agreement with results of McKee and colleagues. It is noteworthy that in Braak stages V–VI, in sections of 7-µm thickness, a net of labeled NTs rather than NFT were seen even with the naked eye in areas 17 and 18. The visual association area, area 19, was in some of our cases partly included but unfortunately, not to the same extent as in the study by McKee and colleagues. Thus, we are unable to adequately compare IHC labeling of NFT in our 30 cases with the results obtained by McKeen and colleagues. Furthermore, the section thickness (7 µm vs. 50 µm) might also alter IHC labeling of NFT seen in our 30 cases when compared with their 41 subjects. Their results are, however, of great interest, as the visual association cortex is certainly easier to harvest and identify when compared with some of the neuroanatomical regions in the anterior hippocampus. Additional studies are urgently needed to confirm these results.

A predictable sequence of progression of lesions facilitates a staging strategy that can be followed when assessing neurodegenerative lesions (3, 5, 17). It should, however, be remembered that the proposed predictable sequence of development of some common brain lesions has recently been challenged; for example, the results of McKee and colleagues are difficult to reconcile with the traditional sequence of neuronal pathology in AD (11). Interestingly, with regard to α-synuclein (αS) pathology, the alteration in the predictable progressions of pathology has been proposed to be due to concomitant AD pathology (16, 22). In line with the above, in familial cases carrying the presenilin-1 mutation, studies with positron emission tomography agent Pittsburgh compound-B, indicate that Aβ deposition begins in the striatum rather than in cortex (10). Furthermore, it has been reported that the apolipopreotein E ε4 allele modifies the deposition of Aβ in vessel walls (15, 18). These recent reports regarding NFT, Aβ, and αS pathology indicate that the progression of pathology does not always follow the proposed sequence and that alternative routes of progression might exist, perhaps because of genetic predisposition, concomitant diseases, brain pathologies or environmental factors. These alterations need to be recognized as they might significantly alter the assessment results.

Neuropathologists need detailed diagnostic instructions as was already shown by the EURAGE study (7). Furthermore, the methods used when visualizing lesions of interest should be reproducible (2) and preferably be specific in that they have a molecular basis. In addition, the methods should be applicable for routine working situations. Based on our results, reliable assessment of one of the culprits in AD namely HP-tau containing lesions in an interlaboratory setting can be reached when the instructions are simple and clear, the IHC labeling is robust and the lesions are substantial, that is, they have spread to isocortical structures. In contrast, in subjects where only mild subtle lesions can be seen (AD-related NT pathology in stage I–III), it is recommended that reassessment of lesions by two independent assessor should be carried out, especially in a research setting.

It should also be kept in mind that biological events are not always strict and thus the proposed sequential development of pathology might be altered by a variety of factors and thus influence the staging results.
